# Social Determinants of Health Information Seeking among Chinese Adults in Hong Kong

**DOI:** 10.1371/journal.pone.0073049

**Published:** 2013-08-23

**Authors:** Man Ping Wang, Kasisomayajula Viswanath, Tai Hing Lam, Xin Wang, Sophia S. Chan

**Affiliations:** 1 School of Public Health, The University of Hong Kong, Hong Kong SAR, China; 2 Center for Community-Based Research, Dana-Farber Cancer Institute/Department of Social and Behavioral Sciences, Harvard School of Public Health, Boston, Massachusetts, United States of America; 3 School of Nursing, The University of Hong Kong, Hong Kong SAR, China; Iran University of Medical Sciences, Iran (Islamic Republic Of)

## Abstract

**Background:**

Health communication inequalities were observed in Western population but less is known about them among the Chinese. We investigated health information seeking behaviours and its social determinants among Chinese adults in Hong Kong.

**Methods:**

Probability-based sample surveys over telephone were conducted in 2009, 2010/11 and 2012 to monitor family health and information use. Frequency of health information seeking from television, radio, newspapers/magazines and Internet were recorded and dichotomised as ≥1 time/month and <1 time/month (reference). Logistic regression was used to yield adjusted odds ratios (aOR) of health information seeking for different demographic characteristics, socioeconomic status (education, employment and income), chronic disease and behaviours (smoking, drinking and physical activity).

**Results:**

Among 4553 subjects in all surveys, most (85.1%) had sought health information monthly from newspapers/magazines (66.2%), television (61.4%), radio (35.6%) or Internet (33.2%). Overall, being male, lower education attainment, lower household income, ever-smoking and physical inactivity were associated with less frequent health information seeking (all P <0.05). Compared with younger people, older people were less likely to search health information from Internet but more like to obtain it from radio (both P for trend <0.001). Having chronic diseases was associated with frequent health information seeking from television (aOR  =  1.25, 95% CI: 1.07–1.47) and Internet (aOR  =  1.46, 95% CI: 1.24–1.73).

**Conclusions:**

This study has provided the first evidence on health information inequalities from a non-Western population with advanced mass media and Internet penetration. Socioeconomic inequalities and behavioural clustering of health information seeking suggested more resources are needed for improving health communication in disadvantage groups.

## Introduction

Social factors play important roles in explaining health disparities and one plausible mechanism is through inequalities in health communication, defined as the difference in accessing, seeking, processing and acting on information by different groups in a society [1], [2]. Mass media and Internet are main sources for health information seeking and their influence is evident across the continuum of health from disease prevention, treatment to recovery or end of life [3]. Benefits of health information, particularly among patients with chronic diseases (e.g. cancer), are well-documented including better compliance, improved self-management, reduced psychological stress and increased satisfaction [4].

Although health information seeking behaviour (HISB) is partly explained by psychological factors (e.g. self-efficacy, trait, perception and belief), increasingly studies report that HISB is socially patterned mostly based on evidence from studies in the United States (US) [4]–[6]. Seeking health information requires both financial resources and cognitive skills developed through schooling which puts those from a lower socioeconomic status (SES) at a greater disadvantage. Health communication behaviours were also influenced by race and ethnicity due to culture difference and language barriers [5]. However, inequalities in health information seeking is less known in Asian populations which, compared with the West, have different SES inequalities, telecommunication infrastructure and perceptions of health. Only one study among Japanese has reported that lower education attainment and family income were associated with less health information seeking [7]. Explosive development of information technologies in recent years provides easier access to health information and thus may bridge inequalities in health communication [8]. However, the complexity of the use and interpretation of content on the Internet may hinder health communication access, seeking and uses among disadvantaged groups.

Health information seeking is also patterned by other demographic and lifestyle factors. HISB, particularly on Internet, is less frequent among the elderly probably due to the higher perceived barriers, poor health literacy and a lack of interest in self-health management [9]. In general, males and non-Hispanic Whites are less likely to seek health information [4]. HISB patterns may vary with lifestyle behaviours, for example, heavy smokers sought health information less frequently than light and moderate smokers [10]. A bidirectional association between health problems and HISB was also reported [9].

Hong Kong (93.6% are Chinese), the most westernised city in China, has a profound and widening income inequalities with a higher Gini Coefficient (from 0.43 in the 1970s to 0.53 in the 2000s) than Japan (0.45 in the 2000s) and other developed countries [11], [12]. Mass media in Hong Kong is vibrant owing to the complete freedom of speech and the universal coverage of television (87.2% watched television for more than one hour per day) and radio broadcasting.[13] Daily newspaper circulation ranks 3^rd^ in Asia and 14^th^ in the world (222 copies per 1000 people).[14] In recent years, newspaper readership has been increasing (69% read newspaper daily) due to a surge in the number of free newspapers.[15] Moreover, advanced cyber-infrastructure and relatively low cost of Internet access in Hong Kong have led to high prevalence of Internet use (72.9% have used Internet in the past 12 months) [16]. Prior local studies have reported that HISB on Internet were associated with being younger female and people with positive perception towards Internet use [17], [18]. These findings may not be generalizable as a non-representative sample was adapted [17] and only Internet users were included [18]. Therefore, we investigated the pattern of health information seeking and the association of social determinants with HISB in mass media and the Internet in a representative sample of Hong Kong Chinese adults.

## Methods

### Ethical Statement

Ethical approval was granted by Institutional Review Board (IRB) of the University of Hong Kong/Hospital Authority Hong Kong West Cluster. Verbal informed consents were obtained from the respondents and the procedure was approved by the IRB.

### Survey design

The Hong Kong Family and Health Information Trends Survey (FHinTs) was a regular survey (about once every 12–18 months), and the first 3 were conducted in 2009 (Nov-Dec), 2010/11 (Dec-Mar) and 2012 (Aug-Oct) using probability-based telephone surveys of the general public to monitor the opinions and behaviours on family health, information use and health communication. All interviews were conducted by trained interviewers of the Public Opinion Programme, The University of Hong Kong. The survey targeted the Cantonese-speaking adult population aged 18+. Interviews were mostly conducted in the evenings (6–10pm) and day time when the respondents could not be reached in the evening. A 2-stage random sampling method was used. Telephone numbers (seed numbers) were retrieved from residential telephone directories which cover about 76% of Hong Kong residents [19]. To capture the unlisted telephone numbers, the last digit of the seed numbers with plus or minus one or two generated new random telephone numbers. The telephone numbers were then listed in a random order by a computer programme. Invalid household numbers, non-response calls (not reachable after 8 times of calling) and ineligible households (non-household telephone number, unable to speak Cantonese or aged <18) were excluded. In the second stage, after interviewers introduced the study purpose, adult respondents were asked how many eligible persons were living in the households. All eligible persons were listed and those with dates of the next birthday closest to the interview days and were immediately available for interviews were selected. Each interview took about 20 minutes to complete.

### Measurement

Frequency of health information seeking was assessed by 4 separate questions: “In the past 12 months, how often have you: watched television, listened to radio, read newspapers/magazines and surfed on Internet for health related information?” with responses of “≥1 time/week”, 1–3 times/month”, “1 time in several months”, “rarely” and “never”. The frequencies were dichotomised as ≥1 time/month (monthly) and <1 time/month (reference). As in other similar studies [5], [7], socioeconomic status (SES) was measured using education attainment, household monthly income and employment. Several studies have documented the influence of these SES variables on a variety of health outcomes [*20*]–[22] *.* The responses for SES and behaviours were based on our previous studies and the Hong Kong census with slight modifications. Education attainment was categorised as primary or below (combining “no formal education” and “primary education”), secondary, and tertiary or above. Monthly household income was categorised as <$10,000 (combining “<$4,000” and “$4,000–$9,999”); $10,000–$19,999; $20,000–$29,999; $30,000–$39,999; and ≥$40,000 (HKD, 1 USD  =  7.8 HKD). Employment status was categorised as full-time, pat-time, self-employed, and unemployed. Smoking was categorised as never-smokers, ex-smokers and current smokers who smoked daily or occasionally. Similarly, alcohol drinking was categorised as never-drinkers, ex-drinkers, occasional drinkers (less than once per month) and monthly drinkers (1–3 days per month) and weekly drinkers (at least 1 day per week). Frequency of moderate physical activities for10 minutes in the past 7 days was classified as none, 1–3 days and 4–7 days. History of doctor-diagnosed chronic diseases (cancer, cardiovascular diseases, respiratory diseases, liver diseases, allergy and others) were recorded and classified as none and any. Other collected information included sex, age and marital status.

### Statistical analysis

Sample representativeness was estimated by comparing basic characteristics with Hong Kong 2011 census data using Cohen’s effect with a smaller size indicating greater representativeness of the samples [23]. All data were weighted by sex and age from the census data. Prevalence of monthly health information seeking by SES was tracked from 2009 to 2012. Inequalities of health information seeking by sex, age, marital status and SES indicators were assessed by logistic regression which yielded adjusted odds ratios (aOR) of health information seeking. Associations of behaviours and chronic diseases with health information seeking were also analysed in a separate model adjusting for socio-demographic characteristics and SES. Sensitivity analysis was conducted using a different cut-off point of health information seeking (ever vs never). All analyses were performed using STATA 10. A P-value of less than 0.05 was considered statistically significant.

## Results

Among 6222 adults with confirmed eligibility, 4553 were successfully interviewed which yielded a response rate of 73.2%. Sex, age and living area distributions of the survey subjects were similar to Hong Kong 2011 census population data (Cohen’s effects were small: 0.02–0.17) suggesting that the sample is quite representative [23]. Of 4553 subjects, 54.1% were female, 74.0% were aged 25–64 and 61.9% were married or cohabitated (Table 1). Most subjects had secondary or above education (75.3%) and 57.1% had monthly family income of $20,000 or above (average monthly income in Hong Kong was $20,200). Among 2012 unemployed subjects (44.2%), mostly were retired (42.3%) or homemakers (33.2%). Only a small proportion of subjects were smokers (9.0%) and weekly alcohol drinker (10.7%), near half (44.0%) were physically inactive and one-third (32.2%) had diagnosed chronic diseases.

**Table 1:** Socio-demographic characteristics, chronic disease and behaviours of all subjects in 3 surveys (%).

		Un-weighted	Weighted
Sex	Male	37.6	45.9
	Female	62.4	44.1
Age	18–24	12.6	10.5
	25–44	23.9	37.6
	45–64	47.1	36.4
	≥65	16.4	15.6
Marital status	Single	27.4	32.0
	Married/cohabitated	65.3	61.9
	Others (divorced/widowed)	7.4	6.1
Education	≤Primary	18.7	14.8
	Secondary	50.3	48.4
	≥Tertiary	31.0	36.9
Employment status	Full-time	37.5	46.0
	Part-time	6.8	6.3
	Self-employed	2.9	3.5
	Unemployed	52.8	44.2
Monthly household income	<$10,000	22.4	19.4
	$10,000–$19,999	24.5	23.5
	$20,000–$29,999	20.7	21.4
	$30,000–$39,999	14.3	14.2
	≥$40,000	21.4	21.4
History of chronic disease	No	64.3	67.8
	Yes	35.7	32.2
Smoking	Never	85.8	84.3
	Ex-smokers	6.5	6.7
	Smoker	7.7	9.0
Drinking	Never	53.8	50.5
	Ex-drinkers	1.5	1.4
	Occasional drinkers	26.7	28.2
	1–3 days/month	8.4	9.3
	1 day+/week	9.6	10.7
Moderate physical activity	None	44.2	44.0
	1–3 days/week	25.0	27.1
	4 days+/week	30.9	29.0

Among all respondents, most (85.1%) had sought health information at least once a month (monthly) from newspapers/magazines (66.2%), television (61.4%), radio (35.6%) or Internet (33.2%) (Table 2). During 2009–2012, monthly health information seeking on television and radio came down slightly from 62.2% to 61.4% and 35.1% to 33.2%, respectively, (all P for trend <0.05). In contrast, prevalence of online health information seeking increased from 30.6% in 2009 to 35.6% in 2012 (P for trend <0.001).

**Table 2:** Prevalence (weighted) of monthly health information seeking (%, 95% CI) by year.

	Year					
Sources	2009	2010	2012	Average	OR (95% CI)†	P for trend‡
Newspaper /magazines	65.5	66.1	67.1	66.2	1.87	0.64
	(63.1–67.9)	(63.7–68.5)	(64.7–69.4)	(32.4–35.2)	(0.93–1.25)	
Televisions	62.2	63.8	58.4	61.4	0.86	<0.001
	(59.7–64.6)	(61.3–66.2)	(56.0–60.9)	(60.0–62.9)	(0.74–0.99)*	
Internet	30.6	38.2	38.0	35.6	1.39	<0.001
	(28.3–33.0)	(35.7–40.6)	(35.6–40.4)	(34.2–37.0)	(1.20–1.61)***	
Radio	35.1	33.7	30.9	33.2	0.83	0.04
	(32.7–37.5)	(31.3–36.1)	(28.6–33.2)	(31.9–34.6)	(0.71–0.96)*	
Any	84.3	86.0	85.0	85.1	1.06	0.44
	(82.4–86.2)	(84.1–87.7)	(83.2–86.8)	(84.1–86.1)	(0.87–1.29)	

† Comparing proportion of monthly health information seeking in 2012 vs 2009.

‡ P for trend of odds ratios (OR) of monthly health information seeking using 2009 as reference group.

* P<0.05, **P<0.01, ***P<0.001.

Female and older subjects were more likely to seek health information although increasing age was associated with infrequent health information seeking on Internet (P for trend <0.001) (Table 3). Compared with education attainment of primary or below, higher aORs (95% CI) of monthly health information seeking from any source were 1.55 (1.16–2.07) for secondary and 1.86 (1.32–2.63) for tertiary education or above (P for trend <0.001). This trend was consistently observed for newspapers/magazines, televisions and Internet. In particular, higher education was strongly associated with monthly health information seeking on Internet (secondary education: aOR  =  4.76, 95% CI: 3.23–7.03; tertiary education: aOR  =  8.00, 95% CI: 5.30–12.06). Similarly, increasing household income was associated with higher odds for monthly health information seeking ( P for trend  = 0.07) particularly for newspapers/magazines (P for trend  = 0.05) and Internet (P for trend <0.001). Unemployed subjects were more likely to seek health information than full-time employed subjects (aOR  =  1.42, 95% CI: 1.10–1.82).

**Table 3:** Associations of socio-demographic characteristics and economic status with monthly health information seeking among all subjects in 3 surveys.

	Adjusted odds ratios (95% CI)^a^				
	Newspapers/magazines	Televisions	Internets	Radio	Any
**Sex**					
Male	1	1	1	1	1
Female	1.48 (1.27–1.71)***	1.22 (1.06–1.41)**	1.45 (1.26–1.67)***	0.93 (0.81–1.08)	1.45 (1.20–1.76)***
**Age**					
18–24	1	1	1	1	1
25–44	1.34 (1.03–1.75)*	1.20 (0.92–1.56)	0.73 (0.56–0.96)*	1.28(0.93–1.77)	1.34 (0.94–1.90)
45–64	1.93 (1.43–2.60)***	1.22 (0.92–1.63)	0.42 (0.31–0.56)***	2.39 (1.70–3.37)***	1.91 (1.29–2.84)**
65+	2.21 (1.55–3.14)***	1.36 (0.97–1.91)	0.17 (0.11–0.26)***	3.08 (2.09–4.54)***	2.22 (1.38–3.58)**
P for trend	<0.001	0.11	<0.001	<0.001	<0.01
**Marital status**					
Single	1	1	1	1	1
Married/cohabitated	1.12 (0.92–1.36)	1.06 (0.86–1.31)	0.88 (0.72–1.06)	1.44 (1.18–1.77)***	0.92 (0.71–1.19)
Others	0.89 (0.63–1.26)	1.06 (0.76–1.47)	0.67 (0.43–1.06)	1.26 (0.90–1.78)	1.03 (0.63–1.68)
**Employment status**					
Full-time	1	1	1	1	1
Part-time	0.97 (0.72–1.30)	1.14 (0.87–1.51)	0.83 (0.61–1.12)	0.94 (0.69–1.27)	1.38 (0.92–2.07)
Self-employed	0.79 (0.56–1.13)	1.31 (0.89–1.94)	1.04 (0.72–1.50)	1.57 (1.11–2.23)**	1.34 (0.81–2.24)
Unemployed	1.03 (0.85–1.23)	1.37 (1.15–1.62)***	1.09 (0.90–1.32)	0.98 (0.81–1.80)	1.42 (1.10–1.82)**
**Education**					
≤Primary	1	1	1	1	1
Secondary	2.12 (1.71–2.62)***	1.09 (0.90–1.32)	4.76 (3.23–7.03)***	1.17 (0.95–1.44)	1.55 (1.16–2.07)***
Tertiary+	2.65 (2.04–3.41)***	1.27 (1.00–1.62)*	8.00 (5.30–12.06)***	1.03 (0.80–1.32)	1.86 (1.32–2.63)***
P for trend	<0.001	0.04	<0.001	0.84	<0.001
**Monthly household income**					
<10,000	1	1	1	1	1
$10,000–$19,999	1.38 (1.11–1.72)**	1.18 (0.96–1.46)	2.00 (1.52–2.64)***	0.96 (0.77–1.20)	1.79 (1.33–2.42)**
$20,000–$29,999	1.50 (1.19–1.90)**	1.39 (1.12–1.74)**	2.44 (1.84–3.23)***	0.83 (0.66–1.05)	1.51 (1.11–2.05)*
$30,000–$39,999	1.40 (1.07–1.83)*	1.17 (0.91–1.50)	2.45 (1.81–3.32)***	0.96 (0.74–1.25)	1.60 (1.13–2.28)*
≥$40,000	1.37 (1.07–1.70)*	1.05 (0.8301.33)	2.23 (1.25–3.00)***	0.84 (0.65–1.09)	1.64 (1.16–2.32)*
P for trend	0.05	0.83	<0.001	0.14	0.07

a Adjusting for year, and all variables in the table were mutually adjusted.

* P<0.05, **P<0.01, ***P<0.001.

Never smokers were more likely to seek health information from any sources (aOR  =  1.39, 95% CI: 1.03–1.88) particularly on Internet (aOR  =  1.48, 95% CI: 1.12–1.95) and radio (aOR  =  1.54, 95% CI: 1.18–2.00), compared with current smokers (Table 4). Compared with physically inactive subjects, engaging in physical activity was associated with monthly health information seeking from television, radio, newspapers/magazines and Internet (all P <0.05). Overall, having chronic diseases were not consistently associated with monthly health information seeking (aOR  =  1.18, 95% CI: 0.95–1.47) except that higher odds for online (aOR  =  1.46, 95% CI: 1.24–1.73) and television (aOR  =  1.25, 95% CI: 1.07–1.47) were observed among subjects with chronic diseases. Alcohol drinking was not consistently associated with monthly health information seeking except that occasional drinkers were more likely to seek health information from newspapers/magazines (aOR  =  1.20, 95% CI: 1.01–1.42) and monthly (1–3 days/month) drinkers were more likely to seek health information on Internet (aOR  =  1.49, 95% CI: 1.16–1.92) and radio (aOR  =  1.39, 95%CI: 1.08–1.78), compared with never drinkers. We repeated all the analyses by using cut-off point of health information seeking as “ever” vs “never”, which yielded similar associations (data not shown).

**Table 4:** Associations of health behaviours and status with health information seeking among all subjects in 3 surveys.

	Adjusted odds ratios (95% CI) a				
	Newspapers/magazines	Televisions	Internets	Radio	Any
**Smoking**					
Current smokers	1	1	1	1	1
Ex-smokers	1.03(0.73–1.45)	0.77 (0.55–1.07)*	1.37 (0.92–2.06)	1.09 (0.76–1.56)	1.11 (0.72–1.70)
Never smokers	1.34 (0.88–1.45)	1.04 (0.82–1.33)	1.48 (1.12–1.95)**	1.54 (1.18–2.00)**	1.39 (1.03–1.88)*
**Drinking**					
Never	1	1	1	1	1
Ex-drinking	0.86 (0.49–1.53)	1.32(0.74–2.38)	1.82 (0.92–3.58)	0.71 (0.39–1.80)	1.18 (0.54–2.62)
Occasional drinking	1.20 (1.01–1.42)*	1.10 (0.94–1.29)	0.99 (0.84–1.18)	0.99 (0.84–1.17)	1.08 (0.86–1.35)
1–3 days/month	1.13 (0.88–1.37)	1.07 (0.84–1.36)	1.49 (1.16–1.92)**	1.39 (1.08–1.78)*	1.24 (0.87–1.75)
Weekly drinking	1.07 (0.85–1.37)	0.99 (0.78–1.24)	0.98 (0.76–1.27)	1.11 (0.88–1.42)	0.94 (0.68–1.28)
**Moderate physical activity**					
None	1	1	1	1	1
1–3 days/week	1.54(1.30–1.83)***	1.84 (1.56–2.17)***	1.66 (1.39–1.97)***	1.45 (1.22–1.72)***	1.97 (1.55–2.49)***
4 days+/week	1.66 (1.41–1.97)***	1.38 (1.17–1.60)***	1.58 (1.32–1.89)***	1.20 (1.01–1.41)*	1.82 (1.44–2.29)***
**Chronic diseases**					
No	1	1	1	1	1
Yes	1.10 (0.93–1.29)	1.25(1.07–1.47)**	1.46 (1.24–1.73)*	1.01 (0.86–1.19)	1.18 (0.95–1.47)

a Adjusting for sex, age, marital status, education, employment, income, year and mutually adjusted for variables in the table.

## Discussion

We have provided the first evidence of SES-based inequalities in health information seeking among Chinese adults in a highly Westernised city in China. Having lower education level and household income were associated with less frequent health information seeking. The findings were consistent with the US study on the inequalities of cancer information seeking and the Japanese study which indentified inequalities in information seeking on Internet [5], [7]. Having higher education attainment and income correlates with health literacy and self-efficacy on information searching, and both are important predictors of health information seeking [24], [25]. Among various sources of health information seeking, the inequalities of SES was particularly strong for searching for health information on the Internet in our study. This is consistent with findings from Western and other local studies [17], [26], and supports the concept of digital divide which posits the differential access to Internet among different social class, racial/ethnic and geographic groups. The increasing use of Internet in Hong Kong may result in wider disparity of health communication given the large difference of health information seeking between socioeconomic groups.

Overall, being female, increasing age, never-smoking and physically active were associated with frequent health information seeking. Alcohol drinking and chronic diseases were not consistently associated with health information seeking. The finding that older people in our study were more likely to seek health information from newspapers/magazines and radio was in contrast to the inverse associations reported in Western studies [9], [27]. This may be due to Asian elderly’s tendency to trust health information from newspapers as observed in Japanese [7]. Traditional mass media remain as an important channel for disseminating simple and direct health education to the elderly while Internet use is relatively rare for older people.

Infrequent health information seeking was also observed for smokers and physically inactive subjects. This is in line with studies which found that health information seekers had a healthier lifestyle than avoiders [28]. Indeed, these people have greater needs for health information to restore healthy behaviors. More studies are needed to investigate the associations between health communication inequalities and risk behaviors.

Health information seeking is more prevalent in Hong Kong (85.1% sought health information at least monthly) compared with Japan where only 26.1% people reported recent health information seeking although using slightly different definition of frequency [7]. The near universal coverage of mass media (television and radio), increasing number of free newspapers (5 major free newspapers with daily circulation of 2 million copies) and high Internet access in Hong Kong provide immense opportunities for health information seeking [19]. Newspapers and magazines remained the common sources of health information probably due to increased popularity of free newspapers. A local survey has found a surge of readership of free newspapers from 27% in 2009 to 36% in 2011 and increase in reading time and number of newspapers [15]. Internet use increased from 70.2% in 2009 to 72.9% in 2012. Using same data, we found stable trends of television screen time (from 2.78 hours/day in 2009 to 2.76 hours/day in 2012) and radio listening time (from 1.28 hours/day to 1.21 hours/day in 2012). Prevalence of Internet use for health information seeking (57.8% sought at least monthly) was comparable with those reported among US adults (61.0% sought health information) [29]. This indicated that Internet is an important platform for facilitating health communication. However, using health information with varying quality on the Internet required high level of comprehension skills [30] which may hinder health information seeking among people with lower socioeconomic status.

Our study has several limitations. First, the temporal sequence of factors and health information seeking was uncertain given the cross-sectional design. It is unlikely that health information seeking would lead to lower education attainment and household income. Nevertheless, health information deficit may result in poor knowledge and perception towards health behaviours thus might be prone to reverse causality. Prospective studies are needed to confirm the findings. Second, our study is the first step to describe the general pattern and factors associated with health information seeking in this under-studied Chinese population. Future studies may explore more detailed information on health communication including attention, process, trust and use of health information. Studies are also needed to examine the quality of health information sought on Internet particularly information in Chinese. Third, information was based on self-reporting and studies using more valid methods in tracking behaviour such as Internet use are warranted. Questions for assessing health information seeking were mostly adapted from the US Health Information National Trends Survey (HINTS). We are not certain about the direction of recall bias, if any, on the association. As an important focus of these surveys was on family information, it was unlikely that respondents or interviewers systematically introduced bias when reporting health information seeking. Although our samples are quite representative to general population, we are not certain about the influence of non-response bias and decrease in landline telephones on the findings.

## Conclusion

This study has provided the first evidence on health information inequalities from a non-Western population with advanced mass media and Internet structures. Socioeconomic inequalities and behavioural clustering of health information seeking suggested more resources may be needed for improving health communication in disadvantage groups.
